# Dietary preference, physical activity, and cancer risk in men: national health insurance corporation study

**DOI:** 10.1186/1471-2407-8-366

**Published:** 2008-12-11

**Authors:** Young Ho Yun, Min Kyung Lim, Young-Joo Won, Sang Min Park, Yoon Jung Chang, Sang Woo Oh, Soon Ae Shin

**Affiliations:** 1 National Cancer Control Research Institute, National Cancer Center, Goyang, Gyeonggi, Korea; 2 Department of Family Medicine, Center for Health Promotion, Mitochondrial Research Group, and Clinical Research Center, DongGuk University International Hospital, Gyeonggi, Korea; 3 National Health Insurance Corporation, Seoul, Korea; 4 Department of Family Medicine, Seoul National University Hospital, Seoul National University College of Medicine, Seoul, Korea

## Abstract

**Background:**

The effects of vegetable preference and leisure-time physical activity (LPA) on cancer have been inconsistent. We examined the effects of dietary preference and physical activity, as well as their combined effect on cancer risk.

**Methods:**

This prospective cohort study included 444,963 men, older than 40 years, who participated in a national health examination program begun in 1996. Based on the answer to the question "What kind of dietary preference do you have?" we categorized dietary preference as (1) vegetables, (2) mixture of vegetables and meat, and (3) meats. We categorized LPA as low (< 4 times/wk, < 30 min/session), moderate (2–4 times/wk, ≥ 30 min/session or ≥ 5 times/wk, < 30 min/session), or high (≥ 5 times/wk, ≥ 30 min/session). We obtained cancer incidence data for 1996 through 2002 from the Korean Central Cancer Registry. We used a standard Poisson regression model with a log link function and person-time offset to estimate incidence and relative risk..

**Results:**

During the 6-year follow-up period, we identified 14,109 cancer cases. Multivariate analysis revealed that a preference for vegetables or a mixture of vegetables and meat as opposed to a preference for meat played a significant protective role against lung cancer incidence (aRR, 0.81; 95% confidence interval [CI], 0.68–0.98). Compared with the low LPA group, subjects with moderate-high LPA had a significantly lower risk for stomach (aRR, 0.91; 95%CI, 0.86–0.98), lung (aRR, 0.83; 95%CI, 0.75–0.92), and liver (aRR, 0.88; 95%CI, 0.81–0.95) cancer. Among current smokers, the combined moderate-high LPA and vegetable or mixture of vegetables and meat preference group showed a 40% reduced risk of lung cancer (aRR, 0.60; 95%CI, 0.47–0.76) compared with the combined low LPA and meat preference group. Among never/former smokers, subjects with moderate-high LPA and a preference for vegetables or a mixture of vegetables and meat showed reduced stomach cancer risk (aRR, 0.72; 95%CI, 0.54–0.95).

**Conclusion:**

Our findings add to the evidence of the beneficial effects of vegetable preference on lung cancer risk and of physical activity on lung, stomach, and liver cancer risk. Additionally, vegetable preference combined with LPA might significantly reduce lung and stomach cancer risk.

## Background

Many studies have investigated the protective effect of vegetable consumption against specific cancers, especially colon,[1,2] prostate,[3] pancreas,[4] bladder,[5] lung,[1,6-8] and stomach cancer.[1,9] Moderate-high levels of physical activity (PA) may also have a protective effect, specifically against breast,[10] colon,[2] prostate,[11] and lung cancer.[12]

Previous studies have suggested that PA may have beneficial impact on reducing the sex hormones or insulin resistance and improving immune system, maintaining a healthy body weight by balancing caloric intake with energy expenditure, which could lead to cancer prevention.[13] Vegetables and fruits are known to contain numerous potentially beneficial anti-oxidants, fibers, minerals, and phytochemicals that may help prevent cancer and contribute to maintenance of a healthy weight.[14]

Recently, American Cancer Society (ACS) has released the recommendation of PA and nutrition for cancer prevention. Their recommendation is "at least 30 minutes or more of moderate to vigorous activity, above usual activities, on 5 or more days of the week" and "eating five or more serving of vegetables and fruits each day".[15]

However, findings of the protective effects of vegetable diet and physical activity on cancer risk from numerous epidemiological studies have been inconsistent[3,6] and only few studies distinguished the effect of vegetables and fruits.[1,8] Moreover, most studies did not simultaneously evaluate the combination effect of diet and physical activity to cancer risk. As dietary intake and physical activity, like tobacco use, are cancer risk factors that can probably be modified through lifestyle changes.[15] The relationship of diet and PA to other cancer risk factors, such as smoking, alcohol, body mass index (BMI), and glucose intolerance, should be considered in attempts to identify the effect of diet and PA on cancer risk.

In this study we evaluated the effects of dietary preference and PA, as well as their combined effect on cancer incidence in a large, population-based Korean male cohort, adjusting for other known risk factors.

## Methods

### Study Population

The National Health Insurance Corporation (NHIC) has provided biannual health examinations which was obligatory by law to government employees and private school faculty members and their dependants. The study subjects derived to the end of 2002, the National Health Insurance Corporation Study (NHICS) in Korea enrolled 1,216,041 (901,979 male) older than 20 years who participated in a national health examination program in 1996.[16] From the cohort, we enrolled 454,691 men older than 40 years, excluding those who already had cancer. We excluded women because the number with cancer was too small for analysis. Finally, 444,963 men who provided complete information on dietary preferences and leisure-time physical activity were left for analysis.

### Data Collection

The NHIC biannual examinations, conducted by medical staff at local hospitals, follow a standardized procedure. The items for test were height, weight, and blood pressure measurements, chest radiography, and urinalysis. Blood sample were also obtained under fasting conditions for blood cell counts and chemistries including serum glucose measurement. Each hospital had internal and external quality control procedures direct by the Korean Association of Laboratory Quality Control. In addition, the full self-administered questionnaires included information regarding medical history, current health status, employment, family history, tobacco and alcohol consumption, dietary preferences, and leisure-time physical activity (LPA). The questionnaire was designed by NHIC and reviewed for reliability, validity, and eligibility by an expert committee, and is regulated by Korean Health Promotion law. Smoking status was classified as current, former, or never smoker on the basis of the response to the following questions: "do you smoke cigarettes now?" When we classified the employment status, white collar included managers, professionals and teachers, and blue collar included skilled workers, service workers, plant and machine operators and elementary occupations.

The incident cancer cases were identified from the Korean Central Cancer Registry data along with the time of diagnosis and type of cancer. As this study involved routinely collected medical data, participant consent was not required. The study was approved by the institutional review boards of the National Cancer Center. The details of the cohort study outcome measures were previously reported.[16]

### Classification of Dietary Preference and Leisure-timePhysical Activity

The information on dietary preference and LPA came from checked-off responses to the initial questionnaire. Based on the answer to "What kind of dietary preference do you have?" we categorized dietary preference as (1) vegetables, (2) mixture of vegetables and meat, and (3) meats. For evaluation of the effects of dietary preference on cancer risk, we reduced the levels to 2 – vegetables or mixture of vegetables and meat, and meat [17]

LPA frequency for vigorous intensity and duration was based on the answers to, "How many times per week do you have vigorous, sweat-producing LPA?" and "How long do you have vigorous, sweat-producing LPA per session?" For LPA frequency, the answer was categorized as (1) none, (2) 1–2 times/wk, (3) 3–4 times/wk, (4) 5–6 times/wk, or (5) every day. For duration of LPA, the answer was categorized as (1) < 30 min/session, (2) 30 min-1 hr/session, (3) 1–2 hr/session, or (4) ≥ 2 hr/session. We classified LPA by combining frequency and duration with vigorous, sweat-producing LPA and the basic ACS concepts as (1) low, ≤ 4 times/wk at < 30 min/session, (2) moderate, 2–4 times/week at ≥ 30 min/session or ≥ 5 times/wk at < 30 min/session, and (3) high, ≥ 5 times/wk at ≥ 30 min/session. For evaluation of the effect of LPA on cancer risk, we reduced the levels of LPA to 2 – low and moderate-high.[15]

When we combined these two health behaviors and evaluated the risk of cancer incidence, we found no difference between the group that had moderate-high LPA and preferred meat and the group that had low LPA and preferred vegetables or a mixture of vegetables and meat. Therefore, for evaluation of combination levels, we used only 3 subgroup categories: (1) least favorable lifestyle: low LPA with meat preference, (2) moderate lifestyle: moderate-high LPA with meat preference or low LPA with vegetable or mixture of vegetables and meat preference, and (3) optimal lifestyle: moderate-high LPA with vegetable or mixture of vegetables and meat preference.

### Statistical Methods

We calculated the person-years at risk for each subject from the date of enrollment to the date of cancer diagnosis or December 31, 2002, whichever came first. We calculated the age-adjusted incidence rates per 100,000 person-years for each cancer site according to dietary preference and LPA. We estimated the adjusted relative risk (aRR) and 95% confidence intervals (CIs) adjusting first for age at enrollment. For multivariate analyses, we adjusted socioeconomic variables such as age and employment (white, blue collar) and the following variables, which are known cancer risk factors: smoking status (current, former, or never smoker), absolute alcohol consumption (< 51.8, 51.8–124.1, 124.2–289.7, ≤ 289.8 g/week), BMI in kg/m^2 ^(< 25, 25–30, ≥ 30), fasting glucose level in mg/dL (< 110, 110–125, ≥ 126), and dietary preference and LPA, as appropriate. We used a standard Poisson regression model, which is suitable for estimating the rate of rare diseases such as cancer, with a log link function and person-time offset to estimate incidence and relative risk.

All statistical tests were two sided and performed with SAS statistical package version 8.1.

## Results

### Baseline characteristics

The mean age of the subjects was 49.0 years (SD, 6.5). During the 6-year follow-up period of 3,124,596.7 person-years, we identified 14,109 cancer cases, including multiple primary cancers.

Table 1 shows the baseline characteristics of the subjects. More subjects with meat preference or low LPA were current smokers, high drinkers, or white collar workers than those with a vegetable or mixed preference or moderate-high LPA. Obese cases, however, were more prevalent in the moderate-high LPA group and the meat preference group.

**Table 1:** Baseline Characteristics of Study Population by Dietary Preference, Leisure-time Physical Activity

Characteristics	Dietary Preference^a^			Leisure-time Physical Activity^b^		
	Meat	Vegetables or mixture of vegetables and meat	*P *value^c^	Low	Moderate-High	*P *value^c^

	(n = 29,518)	(n = 419,614)		(n = 262,146)	(n = 184,781)	

**Age (year)**						

40–49	17,765 (60.18)	236,425 (56.34)		147,574 (56.29)	105,835 (57.28)	

50–59	10,091 (34.19)	154,564 (36.83)	< .001	96,411 (36.78)	66,953 (36.23)	< .001

≥ 60	1,662 (5.63)	28,625 (6.82)		18,161 (6.93)	11,993 (6.49)	

**Employment**						

White collar	7,043 (23.86)	92,669 (22.14)		61,460 (23.44)	37,987 (20.56)	

Blue collar	22,475 (76.14)	326,945 (77.86)	< .001	200,686 (76.56)	146,794 (79.44)	< .001

**Smoking status**						

Never	4,845 (16.66)	94,455 (22.91)		57,139 (22.18)	41,647 (22.86)	

Former	5,184 (17.82)	72,956 (17.70)	< .001	42,178 (16.37)	35,796 (19.65)	< .001

Current	19,057 (65.52)	244,793 (59.39)		158,320 (61.45)	104,716 (57.49)	

**Alcohol, g/wk**						

Never	4,262 (14.64)	63,276 (15.30)		42,791 (16.59)	24,028 (13.15)	

≤ 51.8	4,003 (13.75)	75,773 (18.32)		45,591 (17.68)	33,942 (18.58)	

51.9 – 124.2	6,910 (23.74)	111,724 (27.02)	< .001	65,237 (25.29)	52,933 (28.98)	< .001

124.3 – 289.8	9,375 (32.21)	121,308 (29.33)		76,872 (29.81)	53,438 (29.25)	

≤ 238.9	4,554 (15.65)	41,454 (10.02)		27,424 (10.63)	18,339 (10.04)	

**BMI, kg/m**^2^						

< 25	18,532 (62.81)	290,474 (69.25)		184,343 (69.81)	122,911 (66.54)	

25–29.9	10,418 (35.31)	123,976 (29.56)	< .001	76,647 (29.03)	59,299 (32.10)	< .001

≥ 30	555 (1.88)	5,020 (1.20)		3,067 (1.16)	2,502 (1.35)	

**FBS, mg/dL**						

< 110	25,336 (85.93)	359,506 (85.75)		225,293 (86.03)	157,712 (85.42)	

110–125	2,251 (7.63)	32,619 (7.78)	< .001	20,014 (7.64)	14,624 (7.92)	< .001

≥ 126	1,896 (6.43)	27,130 (6.47)		16,582 (6.33)	12,306(6.66)	

**Leisure-time physical activity**						

Low	18,818 (64.40)	242,023 (58.21)				

Moderate-High	10,401 (35.60)	173,721 (41.79)	< .001			

**Dietary preference**						

Meat				18,818 (7.21)	10,401(5.63)	

Vegetables or mixture of vegetables and meat				242,023 (92.79)	173,721(94.01)	< .001

Abbreviations: BMI, Body Mass Index; FBS, Fasting Blood Sugar

^a ^Dietary preference was categorized as vegetables, mixture of vegetables and meat, and meats.

^b^With the combination of frequency and duration with vigorous, sweat-producing LPA, it was categorized as (1) high; ≥ 5 times/week for ≥ 30 min/time, (2) moderate; 2–4 times/week for ≥ 30 min/time or ≥ 5 times/week for < 30 min/time, and (3) low; ≤ 4 times/week for < 30 min/time or ≤ 1 time/week for ≥ 30 min/time.

^c ^Mantel-Haenzel chi square test for comparison among strata in age, job, smoking status, alcohol amount, BMI, and FBS. Values represent number (%) of subjects

### Dietary preference, LPA and cancer risk

We found a significant protective relationship between vegetable or mixture of vegetables and meat preference and incidence of all cancers combined (RR, 0.92; 95% CI, 0.86–0.99) and of lung cancer (RR, 0.76; 95% CI, 0.63–0.91) in the age-adjusted model. In multivariate analysis, these protective relationships were attenuated slightly but remained statistically significant for lung cancer (RR, 0.81; 95% CI, 0.68–0.98) (Table 2).

**Table 2:** Age-Adjusted Incidence Rate and Relative Risk of Cancer by Dietary Preference, Leisure-time Physical Activity in Korean Men

Cancer site	Dietary Preference^a^		Leisure-time Physical Activity^b^	
	
	Meat	Vegetables or Mixture of vegetables and meat	Low	Moderate-High
**All**	(n = 937)	(n = 12,929)	(n = 8,461)	(n = 5,323)

Age aIR^c^	581.9	536.6	562.6	506.2

Age aRR (95% CI)	1.00	0.92 (0.86 – 0.99)	1.00	0.90 (0.87 – 0.93)

Multivariate (95% CI)^d^	1.00	0.96 (0.90 – 1.03)	1.00	0.91 (0.88 – 0.95)

**Head and neck**	(n = 46)	(n = 644)	(n = 411)	(n = 274)

Age aIR^c^	28.9	27.1	27.7	26.4

Age aRR (95% CI)	1.00	0.94 (0.70 – 1.27)	1.00	0.95 (0.82 – 1.11)

Multivariate (95% CI)^d^	1.00	0.97 (0.72 – 1.31)	1.00	0.96 (0.83 – 1.12)

**Esophagus**	(n = 25)	(n = 268)	(n = 189)	(n = 104)

Age aIR^c^	13.9	9.8	11.1	12.5

Age aRR (95% CI)	1.00	0.70 (0.47 – 1.06)	1.00	0.79 (0.62 – 1.00)

Multivariate (95% CI)^d^	1.00	0.79 (0.53 – 1.20)	1.00	0.84 (0.66 – 1.06)

**Stomach**	(n = 249)	(n = 3,416)	(n = 2,226)	(n = 1,407)

Age aIR^c^	154.0	141.3	147.5	133.3

Age aRR (95% CI)	1.00	0.92 (0.81 – 1.04)	1.00	0.90 (0.85 – 0.97)

Multivariate (95% CI)^d^	1.00	0.95 (0.84 – 1.09)	1.00	0.91 (0.86 – 0.98)

**Lung**	(n = 127)	(n = 1,464)	(n = 1,016)	(n = 558)

Age aIR^c^	78.3	89.4	66.0	94.6

Age aRR (95% CI)	1.00	0.76 (0.63 – 0.91)	1.00	0.79 (0.71 – 0.87)

Multivariate (95% CI)^d^	1.00	0.81 (0.68 – 0.98)	1.00	0.83 (0.75 – 0.92)

**Colorectum**	(n = 106)	(n = 1,729)	(n = 1,076)	(n = 751)

Age aIR^c^	67.4	73.2	73.0	72.9

Age aRR (95% CI)	1.00	1.09 (0.89 – 1.32)	1.00	1.00 (0.91 – 1.10)

Multivariate (95% CI)^d^	1.00	1.10 (0.90 – 1.34)	1.00	0.98 (0.90 – 1.08)

**Liver**	(n = 169)	(n = 2,524)	(n = 1672)	(n = 1,004)

Age aIR^c^	96.7	98.2	104.2	89.1

Age aRR (95% CI)	1.00	1.02 (0.87 – 1.19)	1.00 (ref)	0.85 (0.79 – 0.92)

Multivariate (95% CI)^d^	1.00	1.07 (0.92 – 1.25)	1.00 (ref)	0.88 (0.81 – 0.95)

**Gallbladder**	(n = 26)	(n = 310)	(n = 216)	(n = 122)

Age aIR^c^	16.7	13.2	14.7	11.9

Age aRR (95% CI)	1.00	0.79 (0.53 – 1.18)	1.00	0.81 (0.65 – 1.01)

Multivariate (95% CI)^d^	1.00	0.84 (0.57 – 1.26)	1.00	0.81 (0.65 – 1.02)

**Pancreas**	(n = 21)	(n = 330)	(n = 206)	(n = 143)

Age aIR^c^	13.1	13.5	13.5	13.5

Age aRR (95% CI)	1.00	1.03 (0.66 – 1.61)	1.00	1.00 (0.81 – 1.23)

Multivariate (95% CI)^d^	1.00	1.04 (0.67 – 1.62)	1.00	1.00 (0.81 – 1.24)

**Kidney**	(n = 26)	(n = 368)	(n = 230)	(n = 165)

Age aIR^c^	16.1	15.4	15.4	15.7

Age aRR (95% CI)	1.00	0.95 (0.64 – 1.42)	1.00	1.02 (0.84 – 1.25)

Multivariate (95% CI)^d^	1.00	0.99 (0.67 – 1.48)	1.00	1.01 (0.83 – 1.23)

**Bladder**	(n = 30)	(n = 385)	(n = 251)	(n = 163)

Age aIR^c^	19.0	16.2	16.9	15.7

Age aRR (95% CI)	1.00	0.85 (0.59 – 1.24)	1.00	0.93 (0.76 – 1.13)

Multivariate (95% CI)^d^	1.00	0.90 (0.62 – 1.30)	1.00	0.94 (0.77 – 1.15)

**Prostate**	(n = 19)	(n = 288)	(n = 188)	(n = 117)

Age aIR^c^	10.6	10.2	10.7	9.6

Age aRR (95% CI)	1.00	0.96 (0.60 – 1.53)	1.00	0. 90 (0.72 – 1.14)

Multivariate (95% CI)^d^	1.00	0.95 (0.59 – 1.51)	1.00	0.91 (0.72 – 1.14)

The number in the parenthesis means the number of cancer cases.

Abbreviations: aIR, age adjusted incidence rate; aRR, adjusted relative risk; CI, confidence interval.

^a ^Dietary preference was categorized as meats and vegetables or mixture of vegetables and meat.

^b^With the combination of frequency and duration with vigorous, sweat-producing leisure-time physical activity (LPA), it was categorized as (1) low; ≤ 4 times/week for < 30 min/session or ≤ 1 time/week for ≥ 30 min/session, (2) moderate; 2–4 times/week for ≥ 30 min/session or ≥ 5 times/week for < 30 min/session, and (3) high; ≥ 5 times/week for ≥ 30 min/session

^c ^The rate is per 100,000 person-years, adjusted to the age distribution of the entire study population.

^d^The multivariate relative risk model using standard Poisson regression analysis adjusted for age, dietary preference, LPA, smoking status, amount of alcohol drinking, body mass index, employment and fasting blood sugar as appropriate.

As with the age-adjusted model, multivariate analysis showed that subjects with moderate-high LPA had a significantly lower risk for all cancers combined (RR, 0.91; 95% CI, 0.88–0.95) and for stomach (RR, 0.91; 95% CI, 0.86–0.98), lung (RR, 0.83; 95% CI, 0.75–0.92), and liver (RR, 0.88; 95% CI, 0.81–0.95) cancer (Table 2).

### Dietary preference and cancer risk by smoking status

We found a significant protective association between vegetable or mixture of vegetables and meat preference and risk for lung cancer only in current smokers after adjusting for age (RR, 0.76; 95% CI, 0.63–0.93) and for other potential confounders (RR, 0.77; 95% CI, 0.63–0.95) (Table 3).

**Table 3:** Age-Adjusted Incidence Rate and Relative Risk of Cancer by Smoking Status and Dietary Preference^a^

Cancer site	Never/Former Smokers		Current Smokers	
	
	Meat	Vegetables or mixture of vegetables and meat	Meat	Vegetables or mixture of vegetables and meat
**All**	(n = 275)	(n = 4,586)	(n = 654)	(n = 8,135)

Age aIR^b^	479.0	458.1	653.9	601.9

Age aRR (95% CI)	1.00	0.96 (0.85 – 1.08)	1.00	0.92 (0.85 – 1.00)

Multivariate (95% CI)^c^	1.00	0.97 (0.86 – 1.10)	1.00	0.94 (0.87 – 1.02)

**Head and neck**	(n = 12)	(n = 227)	(n = 34)	(n = 411)

Age aIR^b^	21.2	23.3	34.4	30.7

Age aRR (95% CI)	1.00	1.10 (0.61 – 1.96)	1.00	0.89 (0.63 – 1.26)

Multivariate (95% CI)^c^	1.00	1.11 (0.62 – 2.00)	1.00	0.91 (0.64 – 1.29)

**Esophagus**	(n = 6)	(n = 56)	(n = 19)	(n = 212)

Age aIR^b^	8.9	4.7	17.1	13.8

Age aRR (95% CI)	1.00	0.53 (0.23 – 1.22)	1.00	0.81 (0.51 – 1.29)

Multivariate (95% CI)^c^	1.00	0.56 (0.24 – 1.29)	1.00	0.87 (0.54 – 1.39)

**Stomach**	(n = 82)	(n = 1,171)	(n = 162)	(n = 2,192)

Age aIR^b^	141.6	115.5	161.0	161.6

Age aRR (95% CI)	1.00	0.82 (0.65 – 1.02)	1.00	1.00 (0.86 – 1.18)

Multivariate (95% CI)^c^	1.00	0.83 (0.66 – 1.03)	1.00	1.02 (0.87 – 1.20)

**Lung**	(n = 21)	(n = 343)	(n = 105)	(n = 1,105)

Age aIR^b^	36.8	34.0	104.5	79.8

Age aRR (95% CI)	1.00	0.92 (0.59 – 1.44)	1.00	0.76 (0.63 – 0.93)

Multivariate (95% CI)^c^	1.00	0.95 (0.61 – 1.48)	1.00	0.77 (0.63 – 0.95)

**Colorectum**	(n = 40)	(n = 767)	(n = 66)	(n = 935)

Age aIR^b^	70.3	77.2	68.0	71.1

Age aRR (95% CI)	1.00	1.10 (0.80 – 1.51)	1.00	1.05 (0.81 – 1.34)

Multivariate (95% CI)^c^	1.00	1.12 (0.82 – 1.55)	1.00	1.05 (0.82 – 1.35)

**Liver**	(n = 48)	(n = 921)	(n = 119)	(n = 1,551)

Age aIR^b^	78.8	87.9	107.9	105.9

Age aRR (95% CI)	1.00	1.12 (0.83 – 1.49)	1.00 (ref)	0.98 (0.81 – 1.18)

Multivariate (95% CI)^c^	1.00	1.14 (0.85 – 1.53)	1.00 (ref)	1.02 (0.85 – 1.23)

**Gallbladder**	(n = 10)	(n = 120)	(n = 16)	(n = 185)

Age aIR^b^	18.0	12.4	16.5	14.0

Age aRR (95% CI)	1.00	0.69 (0.36 – 1.31)	1.00	0.85 (0.51 – 1.41)

Multivariate (95% CI)^c^	1.00	0.72 (0.38 – 1.37)	1.00	0.90 (0.54 – 1.51)

**Pancreas**	(n = 6)	(n = 114)	(n = 15)	(n = 210)

Age aIR^b^	9.5	10.2	15.5	15.9

Age aRR (95% CI)	1.00	1.07 (0.47 – 2.43)	1.00	1.02 (0.61 – 1.73)

Multivariate (95% CI)^c^	1.00	1.07 (0.47 – 2.43)	1.00	1.00 (0.59 – 1.70)

**Kidney**	(n = 7)	(n = 142)	(n = 19)	(n = 220)

Age aIR^b^	12.2	14.2	18.9	16.3

Age aRR (95% CI)	1.00	1.17 (0.55 – 2.50)	1.00	0.87 (0.54 – 1.38)

Multivariate (95% CI)^c^	1.00	1.24 (0.58 – 2.64)	1.00	0.89 (0.56 – 1.43)

**Bladder**	(n = 5)	(n = 123)	(n = 25)	(n = 255)

Age aIR^b^	8.7	12.1	25.4	19.1

Age aRR (95% CI)	1.00	1.39 (0.57 – 3.41)	1.00	0.75 (0.50 – 1.14)

Multivariate (95% CI)^c^	1.00	1.41 (0.58 – 3.45)	1.00	0.77 (0.51 – 1.16)

**Prostate**	(n = 8)	(n = 134)	(n = 11)	(n = 142)

Age aIR^b^	12.2	11.2	10.2	9.3

Age aRR (95% CI)	1.00	0.91 (0.45 – 1.87)	1.00	0.91 (0.49 – 1.68)

Multivariate (95% CI)^c^	1.00	0.89 (0.44 – 1.83)	1.00	0.93 (0.50 – 1.72)

The number in the parenthesis means the number of cancer cases.

Abbreviations: aIR, age adjusted incidence rate; aRR, adjusted relative risk; CI, confidence interval.

^a ^Dietary preference was categorized as meats and vegetables or mixture of vegetables and meat.

^b^The rate is per 100,000 person-years, adjusted to the age distribution of the entire study population.

^c ^The multivariate relative risk model using standard Poisson regression analysis adjusted for age, leisure-time physical activity, amount of alcohol drinking, body mass index and fasting blood sugar as appropriate.

### LPA and cancer risk by smoking status

In the analysis of the effect of LPA on cancer risk by smoking status, moderate-high LPA was associated with approximately a 10% reduction in risk of all cancers combined in both never/former and current smokers (Table 4). In the age-adjusted model, current smokers with moderate-high LPA had a significantly lower risk of lung (RR, 0.81; 95% CI, 0.72–0.91) and liver (RR, 0.89; 95% CI, 0.81–0.99) cancer, while never/former smokers with moderate-high LPA showed a significant decrease in stomach (RR, 0.88; 95% CI, 0.78–0.98) and liver (RR, 0.82; 95% CI, 0.72–0.93) cancer risk. These associations were attenuated slightly in multivariate analysis but still remained statistically significant.

**Table 4:** Age-Adjusted Incidence Rate and Relative Risk of Cancer by Smoking Status and Leisure-time Physical activity^a^

Cancer site	Never/Former Smoker		Current Smoker	
	
	Low	Moderate-High	Low	Moderate-High
**All**	(n = 2,839)	(n = 1,995)	(n = 5,494)	(n = 3,257)

Age aIR^b^	478.2	433.6	627.5	570.7

Age aRR (95% CI)	1.00	0.91 (0.86 – 0.96)	1.00	0.91 (0.87 – 0.95)

Multivariate (95% CI)^c^	1.00	0.91 (0.86 – 0.97)	1.00	0.92 (0.88 – 0.96)

**Head and neck**	(n = 125)	(n = 114)	(n = 280)	(n = 157)

Age aIR^b^	21.7	25.4	32.3	27.8

Age aRR (95% CI)	1.00	1.17 (0.91 – 1.51)	1.00	0.86 (0.71 – 1.05)

Multivariate (95% CI)^c^	1.00	1.17 (0.91 – 1.51)	1.00	0.87 (0.71 – 1.05)

**Esophagus**	(n = 37)	(n = 26)	(n = 152)	(n = 78)

Age aIR^b^	5.2	2.5	15.3	12.1

Age aRR (95% CI)	1.00	0.91 (0.55 – 1.50)	1.00	0.79 (0.60 – 1.04)

Multivariate (95% CI)^c^	1.00	0.89 (0.54 – 1.47)	1.00	0.82 (0.62 – 1.08)

**Stomach**	(n = 743)	(n = 505)	(n = 1449)	(n = 886)

Age aIR^b^	123.6	108.5	165.1	154.7

Age aRR (95% CI)	1.00	0.88 (0.78 – 0.98)	1.00	0.94 (0.86 – 1.02)

Multivariate (95% CI)^c^	1.00	0.88 (0.79 – 0.99)	1.00	0.94 (0.86 – 1.02)

**Lung**	(n = 220)	(n = 139)	(n = 788)	(n = 412)

Age aIR^b^	36.8	30.1	87.9	71.0

Age aRR (95% CI)	1.00	0.82 (0.66 – 1.01)	1.00	0.81 (0.72 – 0.91)

Multivariate (95% CI)^c^	1.00	0.82 (0.66 – 1.02)	1.00	0.83 (0.73 – 0.93)

**Colorectum**	(n = 462)	(n = 339)	(n = 600)	(n = 401)

Age aIR^b^	78.4	74.3	70.3	72.3

Age aRR (95% CI)	1.00	0.95 (0.82 – 1.09)	1.00	1.03 (0.91 – 1.17)

Multivariate (95% CI)^c^	1.00	0.93 (0.81 – 1.07)	1.00	1.02 (0.90 – 1.16)

**Liver**	(n = 588)	(n = 375)	(n = 1051)	(n = 616)

Age aIR^b^	94.7	77.6	110.9	99.0

Age aRR (95% CI)	1.00	0.82 (0.72 – 0.93)	1.00	0.89 (0.81 – 0.99)

Multivariate (95% CI)^c^	1.00	0.86 (0.75 – 0.98)	1.00	0.91 (0.82 – 1.00)

**Gallbladder**	(n = 78)	(n = 51)	(n = 133)	(n = 71)

Age aIR^b^	13.6	11.5	15.5	12.7

Age aRR (95% CI)	1.00	0.84 (0.59 – 1.20)	1.00	0.82 (0.62 – 1.10)

Multivariate (95% CI)^c^	1.00	0.85 (0.60 – 1.21)	1.00	0.82 (0.61 – 1.09)

**Pancreas**	(n = 67)	(n = 51)	(n = 134)	(n = 89)

Age aIR^b^	10.1	10.0	15.6	16.0

Age aRR (95% CI)	1.00	0.99 (0.68 – 1.42)	1.00	1.02 (0.78 – 1.34)

Multivariate (95% CI)^c^	1.00	0.96 (0.67 – 1.39)	1.00	1.02 (0.78 – 1.34)

**Kidney**	(n = 84)	(n = 64)	(n = 144)	(n = 97)

Age aIR^b^	14.2	13.9	16.5	17.0

Age aRR (95% CI)	1.00	0.98 (0.71 – 1.36)	1.00	1.03 (0.80 – 1.33)

Multivariate (95% CI)^c^	1.00	0.94 (0.68 – 1.30)	1.00	1.02 (0.78 – 1.32)

**Bladder**	(n = 68)	(n = 60)	(n = 177)	(n = 103)

Age aIR^b^	11.2	12.8	20.4	18.3

Age aRR (95% CI)	1.00	1.14 (0.81 – 1.62)	1.00	0.89 (0.70 – 1.14)

Multivariate (95% CI)^c^	1.00	1.15 (0.81 – 1.63)	1.00	0.89 (0.69 – 1.13)

**Prostate**	(n = 85)	(n = 57)	(n = 97)	(n = 55)

Age aIR^b^	11.9	10.4	9.8	8.7

Age aRR (95% CI)	1.00	0.88 (0.63 – 1.23)	1.00	0.89 (0.64 – 1.24)

Multivariate (95% CI)^c^	1.00	0.89 (0.63 – 1.24)	1.00	0.89 (0.64 – 1.24)

The number in the parenthesis means the number of cancer cases.

Abbreviations: aIR, age adjusted incidence rate; aRR, adjusted relative risk; CI, confidence interval.

^a ^With the combination of frequency and duration with vigorous, sweat-producing leisure-time physical activity (LPA), it was categorized as (1) low; ≤ 4 times/week for < 30 min/session or ≤ 1 time/week for ≥ 30 min/session, (2) moderate; 2–4 times/week for ≥ 30 min/session or ≥ 5 times/week for < 30 min/session, and (3) high; ≥ 5 times/week for ≥ 30 min/session

^b ^The rate is per 100,000 person-years, adjusted to the age distribution of the entire study population.

^c^The multivariate relative risk model using standard Poisson regression analysis adjusted for age, dietary preference, amount of alcohol drinking, body mass index, employment and fasting blood sugar as appropriate.

### Combination effect of dietary preference and LPA on cancer risk

Compared with the least favorable lifestyle group, the optimal lifestyle group showed additive effect as a significantly lower incidence of all cancers combined and lung cancer in both age-adjusted (RR, 0.57; 95% CI, 0.46–0.71) and multivariate-adjusted (RR, 0.63; 95% CI, 0.51–0.79) analysis (Table 5). Similarly, the middle group had significantly lower lung cancer risk in both age-adjusted (RR, 0.69; 95% CI, 0.56–0.86) and multivariate analysis (RR, 0.73; 95% CI, 0.59–0.91) (Table 5). When we divided the subjects by smoking status, for current smokers, the combined effect of moderate-high LPA and vegetable or mixture of vegetables and meat preference on cancer prevention still remained in all cancers combined and in lung cancer. The same was true for the middle group. Interestingly, for never/former smokers, subjects with moderate-high LPA and vegetable or mixture of vegetables and meat preference showed reduced stomach cancer risk in both age-adjusted analysis (RR, 0.71; 95% CI, 0.54–0.94) and multivariate analysis (RR, 0.72; 95% CI, 0.54–0.95).

**Table 5:** Age-Adjusted Incidence Rate and Relative Risk of Cancer by Dietary Preference, Leisure-time Physical Activity^a^, and Smoking Status

Cancer site	Total			Never/Former Smokers			Current Smokers		
	
	Least favorable lifestyle	Middle group	Optimal lifestyle	Least favorable lifestyle	Middle group	Optimal lifestyle	Least favorable lifestyle	Middle group	Optimal lifestyle
**All**	(n = 624)	(n = 8071)	(n = 4997)	(n = 176)	(n = 2746)	(n = 1890)	(n = 445)	(n = 5204)	(n = 3042)

Age aIR^b^	605.2	557.2	504.0	505.9	474.8	433.0	671.3	621.7	567.9

Age aRR (95% CI)	1.00	0.92 (0.85 – 1.00)	0.83 (0.77 – 0.91)	1.00	0.94 (0.81 – 1.09)	0.86 (0.73 – 1.00)	1.00	0.93 (0.84 – 1.02)	0.85 (0.77 – 0.93)

Multivariate (95% CI)^c^	1.00	0.95 (0.88 – 1.03)	0.87 (0.80 – 0.95)*	1.00	0.95 (0.81 – 1.10)	0.87 (0.75 – 1.02)*	1.00	0.94 (0.85 – 1.03)	0.86 (0.78 – 0.95)*

**Head and neck**	(n = 32)	(n = 389)	(n = 259)	(n = 5)	(n = 127)	(n = 107)	(n = 27)	(n = 258)	(n = 150)

Age aIR^b^	31.4	27.3	26.5	14.6	22.6	25.2	41.3	31.1	28.3

Age aRR (95% CI)	1.00	0.87 (0.61 – 1.24)	0.84 (0.58 – 1.22)	1.00	1.54 (0.63 – 3.77)	1.72 (0.70 – 4.22)	1.00	0.75 (0.51 – 1.12)	0.69 (0.46 – 1.03)

Multivariate (95% CI)^c^	1.00	0.90 (0.62 – 1.28)	0.88 (0.61 – 1.27)	1.00	1.57 (0.64 – 3.84)	1.75 (0.71 – 4.29)	1.00	0.76 (0.51 – 1.13)	0.69 (0.46 – 1.05)

**Esophagus**	(n = 15)	(n = 181)	(n = 94)	(n = 4)	(n = 34)	(n = 24)	(n = 11)	(n = 147)	(n = 70)

Age aIR^b^	13.0	11.0	8.4	9.7	4.9	4.6	14.9	15.5	11.6

Age aRR (95% CI)	1.00	0.84 (0.50 – 1.43)	0.64 (0.37 – 1.11)	1.00	0.50 (0.78 – 1.42)	0.47 (0.16 – 1.36)	1.00	1.04 (0.56 – 1.92)	0.78 (0.41 – 1.47)

Multivariate (95% CI)^c^	1.00	0.94 (0.55 – 1.59)	0.75 (0.44 – 1.30)	1.00	0.52 (0.19 – 1.48)	0.48 (0.17 – 1.38)	1.00	1.09 (0.59 – 2.02)	0.85 (0.45 – 1.60)

**Stomach**	(n = 160)	(n = 2132)	(n = 1318)	(n = 53)	(n = 714)	(n = 474)	(n = 104)	(n = 1385)	(n = 830)

Age aIR^b^	154.6	146.7	132.5	151.0	121.9	107.3	156.1	165.0	154.4

Age aRR (95% CI)	1.00	0.95 (0.81 – 1.47)	0.86 (0.73 – 1.50)	1.00	0.81 (0.61 – 1.07)	0.71 (0.54 – 0.94)	1.00	1.06 (0.87 – 1.29)	0.99 (0.81 – 1.21)

Multivariate (95% CI)^c^	1.00	0.98 (0.83 – 1.15)	0.89 (0.76 – 1.05)*	1.00	0.81 (0.61 – 1.07)	0.72 (0.54 – 0.95)*	1.00	1.06 (0.87 – 1.30)	1.00 (0.81 – 1.23)

**Lung**	(n = 95)	(n = 939)	(n = 527)	(n = 14)	(n = 211)	(n = 132)	(n = 81)	(n = 722)	(n = 388)

Age aIR^b^	91.3	63.3	52.1	40.4	36.3	30.1	121.3	84.2	71.1

Age aRR (95% CI)	1.00	0.69 (0.56 – 0.86)	0.57 (0.46 – 0.71)	1.00	0.90 (0.52 – 1.54)	0.75 (0.43 – 1.29)	1.00	0.69 (0.55 – 0.87)	0.59 (0.46 – 0.75)

Multivariate (95% CI)^c^	1.00	0.73 (0.59 – 0.91)	0.63 (0.51 – 0.79)*	1.00	0.91 (0.53 – 1.56)	0.76 (0.44 – 1.32)	1.00	0.69 (0.55 – 0.87)	0.60 (0.47 – 0 76)*

**Colorectum**	(n = 72)	(n = 1,030)	(n = 716)	(n = 28)	(n = 443)	(n = 327)	(n = 44)	(n = 573)	(n = 378)

Age aIR^b^	71.5	72.5	73.7	81.2	77.2	75.5	68.3	70.3	72.6

Age aRR (95% CI)	1.00	1.01 (0.80 – 1.29)	1.03 (0.81 – 1.31)	1.00	0.95 (0.65 – 1.39)	0.93 (0.63 – 1.37)	1.00	1.03 (0.76 – 1.40)	1.06 (0.78 – 1.45)

Multivariate (95% CI)^c^	1.00	1.03 (0.81 – 1.30)	1.03 (0.81 – 1.31)	1.00	0.97 (0.66 – 1.42)	0.94 (0.64 – 1.38)	1.00	1.04 (0.76 – 1.41)	1.07 (0.78 – 1.46)

**Liver**	(n = 108)	(n = 1614)	(n = 936)	(n = 32)	(n = 570)	(n = 358)	(n = 76)	(n = 1009)	(n = 568)

Age aIR^b^	96.7	104.5	88.2	86.9	94.2	78.2	104.4	111.4	97.4

Age aRR (95% CI)	1.00	1.08 (0.89 – 1.31)	0.91 (0.75 – 1.11)	1.00	1.08 (0.76 – 1.55)	0.90 (0.63 – 1.29)	1.00	1.07 (0.85 – 1.35)	0.93 (0.73 – 1.19)

Multivariate (95% CI)^c^	1.00	1.13 (0.93 – 1.37)	0.98 (0.80 – 1.20)	1.00	1.10 (0.77 – 1.56)	0.95 (0.66 – 1.37)	1.00	1.10 (0.87 – 1.39)	0.98 (0.77 – 1.24)

**Gallbladder**	(n = 16)	(n = 207)	(n = 110)	(n = 5)	(n = 78)	(n = 45)	(n = 11)	(n = 124)	(n = 65)

Age aIR^b^	16.0	14.7	11.4	14.9	14.0	10.7	17.1	15.1	12.4

Age aRR (95% CI)	1.00	0.92 (0.55 – 1.52)	0.71 (0.42 – 1.20)	1.00	0.94 (0.38 – 2.32)	0.72 (0.29 – 1.81)	1.00	0.88 (0.48 – 1.64)	0.73 (0.38 – 1.38)

Multivariate (95% CI)^c^	1.00	0.95 (0.58 – 1.60)	0.75 (0.44 – 1.27)*	1.00	0.97 (0.39 – 2.39)	0.74 (0.29 – 1.87)	1.00	0.92 (0.50 – 1.71)	0.75 (0.40 – 1.43)

**Pancreas**	(n = 12)	(n = 200)	(n = 134)	(n = 3)	(n = 67)	(n = 48)	(n = 9)	(n = 130)	(n = 83)

Age aIR^b^	11.7	13.6	13.4	7.9	10.4	9.9	14.0	15.8	15.9

Age aRR (95% CI)	1.00	1.17 (0.65 – 2.09)	1.15 (0.64 – 2.07)	1.00	1.32 (0.42 – 4.21)	1.26 (0.39 – 4.04)	1.00	1.13 (0.58 – 2.22)	1.13 (0.57 – 2.26)

Multivariate (95% CI)^c^	1.00	1.18 (0.66 – 2.11)	1.16 (0.64 – 2.10)	1.00	1.30 (0.41 – 4.13)	1.21 (0.38 – 3.89)	1.00	1.11 (0.57 – 2.19)	1.11 (0.56 – 2.22)

**Kidney**	(n = 18)	(n = 217)	(n = 157)	(n = 4)	(n = 82)	(n = 61)	(n = 14)	(n = 133)	(n = 92)

Age aIR^b^	17.4	15.1	15.9	11.5	14.2	14.0	21.0	15.9	17.2

Age aRR (95% CI)	1.00	0.86 (0.53 – 1.40)	0.91 (0.56 – 1.49)	1.00	1.24 (0.45 – 3.38)	1.22 (0.44 – 3.36)	1.00	0.76 (0.44 – 1.32)	0.82 (0.47 – 1.44)

Multivariate (95% CI)^c^	1.00	0.90 (0.56 – 1.46)	0.94 (0.57 – 1.53)	1.00	1.31 (0.48 – 3.57)	1.24 (0.45 – 3.42)	1.00	0.78 (0.45 – 1.36)	0.83 (0.47 – 1.46)

**Bladder**	(n = 16)	(n = 248)	(n = 149)	(n = 3)	(n = 66)	(n = 58)	(n = 13)	(n = 176)	(n = 91)

Age aIR^b^	15.8	17.3	15.2	8.6	11.2	13.1	19.9	21.2	17.2

Age aRR (95% CI)	1.00	1.10 (0.66 – 1.82)	0.96 (0.58 – 1.61)	1.00	1.31 (0.41 – 4.16)	1.53 (0.48 – 4.88)	1.00	1.07 (0.61 – 1.88)	0.87 (0.48 – 1.55)

Multivariate (95% CI)^c^	1.00	1.15 (0.69 – 1.91)	1.02 (0.61 – 1.71)	1.00	1.32 (0.42 – 4.21)	1.56 (0.49 – 5.00)	1.00	1.08 (0.62 – 1.90)	0.86 (0.48 – 1.55)

**Prostate**	(n = 10)	(n = 187)	(n = 108)	(n = 5)	(n = 83)	(n = 54)	(n = 5)	(n = 98)	(n = 49)

Age aIR^b^	8.7	11.0	9.4	12.5	11.9	10.4	7.0	10.3	8.2

Age aRR (95% CI)	1.00	1.27 (0.67 – 2.40)	1.08 (0.57 – 2.07)	1.00	0.96 (0.39 – 2.36)	0.83 (0.33 – 2.08)	1.00	1.49 (0.61 – 3.65)	1.18 (0.47 – 2.97)

Multivariate (95% CI)^c^	1.00	1.24 (0.66 – 2.35)	1.07 (0.56 – 2.04)	1.00	0.94 (0.38 – 2.31)	0.82 (0.33 – 2.06)	1.00	1.51 (0.61 – 3.71)	1.20 (0.48 – 3.02)

The combination of dietary preference and leisure-time physical activity (LPA) were categorized as (1) the least favorable lifestyle; low LPA with meat preference, (2) middle group; moderate-high LPA with meat preference or low LPA with vegetable or mixture of vegetables and meat preference, and (3) optimal lifestyle; moderate-high LPA with vegetable or mixture of vegetables and meat preference.

The number in the parenthesis means the number of cancer cases.

Abbreviations: aRR, adjusted relative risk; CI, confidence interval

* P Value for trend < 0.05

^a ^With the combination of frequency and duration with vigorous, sweat-producing LPA, it was categorized as (1) low; ≤ 4 times/week for < 30 min/session or ≤ 1 time/week for ≥ 30 min/session, (2) moderate; 2–4 times/week for ≥ 30 min/session or ≥ 5 times/week for < 30 min/session, and (3) high; ≥ 5 times/week for ≥ 30 min/session

^b ^The rate is per 100,000 person-years, adjusted to the age distribution of the entire study population.

^c ^The multivariate relative risk model using standard Poisson regression analysis adjusted for age, amount of alcohol drinking, body mass index, employment and fasting blood sugar as appropriate.

## Discussion

We analyzed the relationship between dietary preference and LPA and the incidence of major cancers among men older than 40 years from the National Health Insurance Corporation Study with a follow-up period of 3,124,596.7 person-years. Of these, 14,109 cases had been diagnosed with at least one primary cancer. This is one of the largest cohort studies to examine such associations and the first to examine the combined protective effects of diet preference and LPA on cancers, including lung, stomach, and liver cancer. We adjusted for age, smoking, and BMI and added job, fasting serum glucose level, and absolute alcohol consumption for multivariate analysis.

Our study showed that vegetables preference was inversely associated with lung cancer risk and that LPA was protective against stomach, lung, and liver cancer. To our knowledge, this is the first prospective cohort study suggesting that moderate to high LPA might be associated with a lower risk of stomach cancer. Our findings also suggested that the combination of both behaviors (vegetable preference and LPA) was more protective, especially against stomach cancer among never/past smokers and lung cancer among current smokers.

Lung cancer was the second common cancer for men in Korea. [18] Although the major risk factor for lung cancer is tobacco smoking, diet and physical activity [10,12] can modify the risk. While some cohort studies found a clear inverse association between vegetable intake and the risk of lung cancer in men,[8] others showed little or no association.[6,7] In some studies, a reduced lung cancer risk was associated not with intake of vegetables, but with intake of fruits.[7] Here we showed that vegetable preference was inversely associated with the risk of lung cancer, especially among current smokers, which is in agreement with other studies,[7,8] but not among former or never smokers in multivariate analysis. The Netherlands Cohort Study also found no significant protective effect of vegetables in never smokers.[8]

Our finding that more regular and intense LPA helped prevent lung cancer, especially among current smokers, is consistent with other studies suggesting that physically active individuals have a lower risk of lung cancer.[3,12] Interestingly, the combination group of both positive behaviors showed an additive effect in current smokers to reduce lung cancer risk 67%. As smoking history is the most important risk factor for lung cancer, we performed an additional analysis adjusting for pack-years of cigarette smoking (< 20, 20–29, 30–39, ≥ 40) in current smokers. That produced only minor changes in the RR of lung cancer associated with dietary preference (RR, 0.83; 95% CI, 0.67–0.97) or with LPA (RR, 0.84; 95% CI, 0.74–0.95). Thus, our study suggests that vegetable preference and regular and intense LPA could be employed together with smoking cessation to reduce the risk of lung cancer.

We found that physically active men, compared with inactive men, had a modest reduction in the risk of developing stomach cancer which is most common cancer in Korean men.[18] This protective effect was found in never/past smokers but not in current smoker. Little information exists on the relationship between physical activity and risk of stomach cancer, and our results are inconsistent with the findings of a case-control study in which stomach cancer was not associated with the Total Activity Index.[19]

Though chronic infection with Hepatitis C virus (HCV) or Hepatitis B virus (HBV) is a major risk factor for liver cancer,[20] several studies suggest that the consumption of vegetables or miso soup is significantly associated with an increased risk of liver cancer.[20] While vegetable preference was not associated with liver cancer risk in our study, moderate or high LPA reduced the risk slightly (RR = 0.88, 95% CI: 0.81–0.95). The protective effect of LPA against liver cancer did not seem to depend on smoking status, even if it was not significant in current smoker. We cannot exclude the possibility of a confounding effect for chronic liver disease, i.e., that subjects with chronic liver disease, a risk of liver cancer, reduced their PA. We had no data on infection status of hepatitis virus. However, this is the first prospective study to report that physical activity may reduce liver cancer risk, suggesting that further study is called for.

In our study, there was no significant association between low LPA or meat preference and colorectal cancer risk. ACS recommends increasing PA and vegetables intake for the colorectal cancer prevention,[15] however, this guideline depended on only two studies.[21,22] Although several studies[1,2,22] also supported a significant reduction in the risk of colon cancer associated with vegetable consumption and PA but it was not consistent.[3,17] Furthermore, recent large prospective cohort studies do not support the hypothesis that PA or vegetables intake is related to a lower incidence of colon cancer.[2] The heterogeneous results may come from the difference of study design or confounding factors.[1] Although our study has the advantage that other important cancer risk factors, such as smoking, alcohol, BMI, and glucose intolerance were considered in the analysis, the occupational physical activity (OPA) total caloric or specific nutritional intake were not collected.

Until now, especially for PA, there is no definited the cut-off level in guideline to prevent the cancer incidence.[15,24] Our classifcation of moderate-high LPA based on frequency and duration of sweat- producing intensity is arbitrary and is different from frequency and duration of LPA recommended in the ACS or World Health Organization (WHO) guidelines on physical activity for cancer prevention.[15,24] When We inferred the intensity of PA from this questionnaire based on the British Columbia physical activity guidelines, one of self-gauging rating factors of intense LPA was "sweat-producing" and "vigorous, sweat-producing LPA" could include the type of LPA such as jogging (7 Metabolic Equivalence Task (MET) per hour(hr)) and so on.[25] With the assumption that "vigorous, sweat-producing LPA" was 7 MET/hr, we could calculated the MET-hr/wk of our LPA category as follow: low LPA, < 10.5 MET-hr/wk; moderate LPA, 10.5–17.5 MET-hr/wk; and high LPA, ≥ 17.5 MET-hr/wk. Recent studies have shown that high physical activity at least 18 MET-hr/wk, which was similar to the category of high LPA in our study, was associated with decrease in colon cancer recurrence and mortality.[26] Another study also suggested that physical activity at least 9 MET-hour/week, which was similar to the category of moderate LPA, might lower the risk of death in breast cancer patients.[27] However, these studies focused on the survival benefit of PA in cancer patients, and further well-designed studies was needed to clarify the cut-off level of PA to prevent the cancer incidence.

Although our study showed that moderate LPA is significantly related with reducing the risk of lung, stomach and liver cancer, higher level of PA could be required to reduce the colorectal cancer risk, as the level of PA to show the survival benefit in colorectal cancer patients[26] was higher than that of breast cancer patients.[27] However, the proportion of subjects with high LPA (≥ 17.5 MET-hr/wk) was few, and the colon cancer incidence of Korea very lower than that of US,[18] which could lead to dilute the real associations. Continued monitoring and long-term follow-up study of our cohort is needed to clarify these associations.

Data on the role of vegetable intake and physical activity in preventing other cancers (e.g., head and neck, pancreas, gall bladder, kidney, and bladder cancer) are limited.[1] Vegetable consumption was not associated with a lowered risk of head and neck cancer in this study, regardless of smoking status. Many case-control studies suggest that fruit, but not vegetables, have a significant protective effect against head and neck cancer.[1,28] In our study, LPA was not significantly associated with a lower risk of head and neck cancer in current smoker. The combination of both behaviors showed similar results. Comparison with other findings is limited by the absence of prospective studies of both behaviors on the risk of head and neck cancer, and the association warrants further investigation.[1]

While neither vegetable preference nor LPA was associated with gall bladder cancer risk, further studies need to be done for LPA because there have been inconsistent findings.[17] Evidence for an association between physical activity and vegetable intake and risk of pancreatic cancer is not consistent. Some case-control study suggest that physical activity and vegetable intake are associated with reduced risk[3] but that was not suggested in our cohort study or a recent prospective study.[29] Thus, the association between vegetable consumption and physical activity and pancreatic cancer risk warrants further investigation.[28]

While smoking avoidance is the most important behavior that can reduce cancer risk,[1] the daily consumption of vegetables or regular physical activity might also reduce risk, especially for lung, stomach and liver cancers. Our findings support the increase of vegetable preference and physical activity as powerful public health measures to reducing cancer[1] and its considerable burden on society.[12]

Our study has several limitations. First, there was a possibility of selection bias. In the study population, only government employees/teachers were included and all women were eliminated, which might have limitation of generalization. In addition, Korean diet is different from North American diet, these cultural differences should be considered to interpret our results. Second, calculating exact MET-hr/wk for LPA was impossible in this study for comparison with other studies,[26,27] because the questionnaire included only about the frequency and duration of sweat-producing activity without specific activity type or intensity. Third, we could not collect the information about OPA or usual activity including household. In multivariate analysis, however, we adjusted for employment status, which might be a surrogate for OPA. In addition, when we stratified the subjects based on employment status, the results were similar to those for the whole population (data not shown). Only the association between LPA and stomach cancer (RR, 0.96; 95% CI, 0.82–1.12) was attenuated and not statistically significant in white collar workers. Forth, we did not collect the diet information in detail. Therefore, we could not analyze the differences in food consumptions between the three diet preferences groups based on study participant's opinion into which group he or she belongs to.

The consumption of common foods over the course of a year, however, is not encoded as a series of discrete episodic events, but preference is inferred from the "liking" of a specific diet effect on attitude, taste factors, and habit. [30] Further study is needed to clarify the association between reported dietary preference and actual food consumed. Fifth, there is a weak scientific evidence for the classification of dietary preferences as (1) meat, (2) vegetable or mixture of vegetables and meat. However, when we performed the analysis with various classifications, we couldn't find significant association between other classification of diet preference and cancer risk. Moreover, one Japanese study also showed that the combined intakes of meat and vegetables on colon cancer incidence.[17]

## Conclusion

To our knowledge, this is first study for dietary preference and cancer risk. As few is known about the association between dietary preference and cancer risk, further studies are needed. Despite these limitations, our findings add to the evidence of the beneficial effects of vegetable preference on lung cancer risk and of physical activity on lung, stomach, and liver cancer risk. This study suggested that the additive effect of combined vegetable preference and LPA might reduce lung and stomach cancers.

## Abbreviations

LPA: leisure-time physical activity; aRR: adjusted relative risk; PA: physical activity; ACS: American Cancer Society; BMI: body mass index; OPA: occupational physical activity; WHO: World Health Organization; MET: Metabolic Equivalence Task.

## Competing interests

The authors declare that they have no competing interests.

## Authors' contributions

YHY conceived of the study and participated in designing it, analyzing and interpreting the data, and drafting the manuscript. SMP and YJC participated in designing the study, analyzing and interpreting the data, and drafting the manuscript. MKL and YJW participated in designing the study, collecting, assembling, and analyzing the data, and drafting the manuscript. SAS participated in designing the study, collecting and assembling the data, and drafting the manuscript. SWO participated in designing the study and drafting the manuscript. All authors read and approved the final manuscript.

## Pre-publication history

The pre-publication history for this paper can be accessed here:


